# Geographic environments, daily activities and stress in Luxembourg (the FragMent study): a protocol combining map-based questionnaires, geographically explicit ecological momentary assessment and vocal biomarkers of stress

**DOI:** 10.1136/bmjopen-2025-105499

**Published:** 2025-09-02

**Authors:** Camille Perchoux, Noemie Topalian, Sylvain Klein, Basile Chaix, Marion Tharrey, Christina Röcke, Philippe Gerber, Olivier Klein, Allyson Missling, Hichem Omrani, Marco Helbich, Delfien Van Dyck, Yan Kestens, Martin Dijst, Guy Fagherazzi

**Affiliations:** 1 LISER, Esch-sur-Alzette, Luxembourg District, Luxembourg; 2 Luxembourg Institute of Health, Strassen, Luxembourg; 3 Institut Pierre Louis d’Épidémiologie et de Santé Publique, Sorbonne Université, Paris, France; 4 Healthy Longevity Center / Center for Gerontology, UZH, Zürich, Switzerland; 5 Human Geography and Spatial Planning, Universiteit Utrecht, Utrecht, The Netherlands; 6 Department of Movement and Sports Sciences, Ghent University, Ghent, Belgium; 7 Centre de recherche en santé publique, Université de Montréal, Montreal, Québec, Canada; 8 University of Luxembourg, Esch-sur-Alzette, Luxembourg

**Keywords:** Stress, Psychological, MENTAL HEALTH, Wearable Devices, Stress, Physiological

## Abstract

**ABSTRACT:**

**Introduction:**

Stress is nearly ubiquitous in everyday life; however, it imposes a tremendous burden worldwide by acting as a risk factor for most physical and mental diseases. The effects of geographic environments on stress are supported by multiple theories acknowledging that natural environments act as a stress buffer and provide deeper and quicker restorative effects than most urban settings. However, little is known about how the temporalities of exposure to complex urban environments (duration, frequency and sequences of exposures) experienced in various locations – as shaped by people’s daily activities – affect daily and chronic stress levels. The potential modifying effect of activity patterns (ie, time, place, activity type and social company) on the environment–stress relationship also remains poorly understood. Moreover, most observational studies relied quasi-exclusively on self-reported stress measurements, which may not accurately reflect the individual physiological embodiment of stress. The FragMent study aims to assess the extent to which the spatial and temporal characteristics of exposures to environments in daily life, along with individuals’ activity patterns, influence physiological and psychological stress.

**Methods and analysis:**

A sample of 2000 adults aged 18–65 and residing in the country of Luxembourg completed a traditional and a map-based questionnaire to collect data on their perceived built, natural and social environments, regular mobility, activity patterns and chronic stress at baseline. A subsample of 200 participants engaged in a 15-day geographically explicit ecological momentary assessment (GEMA) survey, combining a smartphone-enabled global positioning system (GPS) tracking and the repeated daily assessment of the participants’ momentary stress, activities and environmental perceptions. Participants further complete multiple daily vocal tasks to collect data on vocal biomarkers of stress. Analytical methods will include machine learning models for stress prediction from vocal features, the use of geographic information systems (GIS) to quantify dynamic environmental exposures in space and time, and statistical models to disentangle the environment–stress relationships.

**Ethics and dissemination:**

Ethical approval (LISER REC/2021/024.FRAGMENT/4-5-9-10) was granted by the Research Ethics Committee of the Luxembourg Institute of Socio-Economic Research (LISER), Luxembourg. Results will be disseminated via conferences, peer-review journal papers and comic strips. All project outcomes will be made available at https://www.fragmentproject.eu/.

**Strengths and limitations of this study**

- The FragMent study investigates at refined spatial and temporal scales the stressful and restorative qualities of contrasting urban environments.
- This study will use a geographically explicit ecological momentary assessment design, combined with global positioning system tracking and traditional and map-based questionnaires to assess individuals’ exposures along their daily and regular mobility.
- The 15-day follow-up includes four to six measures of stress per day on a sample of 200 adults residing in Luxembourg.
- FragMent will couple self-reported psychological stress measurement with the identification of physiological manifestations of stress based on vocal biomarkers.

## Introduction

### Background and rationale

 Everybody experiences stress. Stress has been defined by the WHO as ‘a state of worry or mental tension caused by a difficult situation’.^1^ It is often expressed as a natural reaction that prompts individuals to face a challenge or a threatening situation: hence the well-known flight-or-fight response.^2^ In practice, stress response can occur in seconds, and it is embodied at psychological, physiological and behavioural levels. The frequency and the duration at which individuals are exposed to stressors and how they respond to them may significantly affect their health.^3^ Indeed, stress is a risk factor of 75% to 90% of diseases,^4^ including psychological, cognitive, mental, cardiovascular, immune, cutaneous, gastrointestinal and respiratory conditions. The prevalence of chronic stress has been rising over the past few decades in low- and middle-income countries, while remaining relatively stable—yet high—at around 36% in high-income countries.^5^ Identifying and addressing modifiable determinants of stress at their roots is therefore a priority.

According to the Stress Reduction Theory^6^ and the Attention Restoration Theory,^7^ the environments in which individuals live and travel as a result of their daily activities play a role in shaping stress levels. Greenspaces are the most extensively researched environmental factor and exhibit strong evidence of inverse associations with physiological and psychological stress.^7^,^10^ However, the identification of stressors and restorative characteristics in complex urban environments remains limited and includes transport and transport infrastructure,^11^ traffic intensity,^12^ noise,^13^,^15^ pollution,^16 17^ accessibility and diversity of amenities, physical decay and disorder,^12 18 19^ neighbourhood deprivation,^20^,^22^ neighbourhood social cohesion^1223^,^26^ and neighbourhood aesthetics.^26 27^

With few exceptions,^13 14 16 28^ observational studies on stress have focused on residential-based environmental exposures. However, daily mobility acts as a ‘vector of exposure’ to various and diverse geographic environments.^29^ There is a need to account for individuals’ activity space, representing the subset of locations visited in the course of daily activities,^30^ to comprehensively estimate the interactions between people, environments and stress.^31^ This may be particularly important for studying social inequalities in stress, as socio-economically disadvantaged groups may experience a double burden by being exposed to more stressful environments both in their residential neighbourhoods and in their non-residential activity spaces.^27^ Furthermore, the visited locations in an activity space may not equally contribute to the environmental effects on stress.^32^ Controlling for individual characteristics, the environment–stress relationship may vary based on the locations visited .^14 28^ One study found a protective effect of greenspaces on psychological stress for all locations except the place of residence,^28^ suggesting that the locations visited embody additional place dimensions (ie, activity type) that modify environment–stress relationships.^33^ Indeed, the type of activity performed – and the social company during these activities – have been hypothesised to ‘modify the conditions of exposure’ to the surrounding environment, thus modifying its effect on stress.^34^

Furthermore, the temporality of exposures, defined by their frequency, duration and sequence over a definite period, has been largely overlooked and simplified when assessing environmental effects on stress.^13 35^ The traditional neighbourhood approaches average environmental effects over the participant’s activity space without taking into account the temporality of these exposures,^36^ or examine stress as it occurs without considering prior or overall space–time exposure conditions.^37^ However, evidence suggests that performing different activities at different times of the day can modify the perception of environmental stressors, mood and psychological stress.^14 38 39^ For example, conducting some activities at 20:00 on a weekday may convey less stress than doing them at other times.^14^ The minimum threshold duration of exposure to limit or induce stress is also factor-dependent. For instance, the relaxing effect of green spaces is greater for exposures lasting between 20 and 30 min, after which benefits continue to increase at a slower rate.^40^ By contrast, noise exposure has an immediate effect on stress.^13^ Finally, the sequence of exposure episodes may theoretically matter^31 34^: the restorative qualities of a given environment could counteract the stressful effect of prior exposures. The rare studies that explore the effect of temporality in exposures on stress, mood or anxiety mostly target exposures to natural environments.^40^,^42^ The lack of systematic testing of multiple temporal configurations of exposures for various environmental factors prevents the development of a clear understanding of ‘what time means for exposures’,^43^ and whether it has an influence on stress.

A last limitation in existing evidence from observational studies is the quasi-exclusively self-reported nature of stress measurements. Perceived stress, whether chronic or momentary, is part of a subjective and cognitive appraisal of a situation,^44^ which may not systematically match the individual’s physiological stress response.^45^,^47^ Beyond self-report, numerous physiological stress responses — variations in cortisol, heartbeat, breath, sweat production, skin temperature and voice can be sensed in real-life observational studies.^34^ However, the data-intensive nature of such (multi)sensor approaches may come with multiple challenges and shortcomings, including a high participation burden, battery and memory limitations of the sensors, modifications of participants’ behaviours (ie, Hawthorne effect), eventual lack of validation of physiological stress measures in outdoor environments and ultimately small sample size due to the reluctance of participation and limited data collection capacities.^34^ Nonetheless, an integrated approach to psychological stress measurement with mobile sensing of physiological stress responses remains the only avenue to comprehensively monitor the complex interplay between environments, behaviours and stress in daily life.^35^

### Objectives and hypotheses

The FragMent project (Geographic environment, daily activities, and stress: a study on the space-time fragmentation of exposure patterns) aims to evaluate the extent to which the spatiality and temporality of exposures to environments in daily life influences physiological and psychological stress, as well as social inequalities therein. The following sub-objectives will be investigated: (i) identify the characteristics of urban environments that are associated with momentary, daily and chronic stress; (ii) define exposure patterns based on the spatiality and temporality of exposure episodes, and assess the associations between of exposure patterns and stress; (iii) investigate the extent to which the effects of exposure patterns on stress are modified by activity patterns and transport modes; (iv) examine the moderating effect of social factors, including gender and socio-economic status, on the relationship between exposure patterns and stress.

To overcome previous limitations, FragMent develops an observational study combining a traditional web-based survey with a map-based questionnaire and a 15-day geographically explicit ecological momentary assessment (GEMA) follow-up to investigate the environmental determinants of momentary, daily and chronic stress. Map-based surveys have proven to be accurate to measure regular mobility patterns,^29 48^ grasping up to 85,5% of global positioning system (GPS) points representing individuals’ actual roaming spaces over 7 days.^49^ Regular mobility and associated environmental exposure measures will be investigated in relation to chronic stress. The GEMA approach will ensure a repeated measurement of participants’ momentary stress, which, combined with continuous mobility measurement, will allow an in-depth investigation of temporal and spatial variations in the environment–stress relationship, accounting for within and between-days variabilities. The study further relies on a combination of self-reported psychological stress measurements and objective measures of physiological manifestations of stress based on vocal biomarkers. Indeed, while stress is a recognised risk factor for developing vocal symptoms,^50^ capturing vocal biomarkers of stress is non-invasive, convenient to collect and less biased than questionnaires that monitor stress.^51^ Stress-induced vocal symptoms may include the that voice becomes strained, tired, hoarse, low in pitch, has voice breaks, throat clearing or coughing and a sensation of a lump in the throat. Vocal biomarkers of stress have been validated against other physiological and psychological stress measures,^5052^,^55^ with accuracy rates as stress or non-stressed classification ranging from 83.7% up to 98% in the latest studies.^56^ Smartphone-based self-assessed stress in real-world environments and during everyday activities has further been correlated with voice features.^57 58^ FragMent is the first study to build on the emergence of vocal biomarkers as a reliable approach to monitor physiological stress in daily life environments in a repeated sampling study design with continuous GPS tracking.

## Methods and analysis

The data collection takes place between the last quarter of 2024 and the third quarter of 2025 in the country of Luxembourg. The survey design includes two participation options. At a minimum, participants complete an online survey (partial participation option) including one traditional web-based questionnaire on their perceived neighbourhood environment and chronic stress and a map-based questionnaire on their regular mobility and activity patterns. In addition, participants can opt to instal an app on their mobile device or request a smartphone from the study team to complete a 15-day mobile survey (full participation option) consisting of a GEMA of activities, perceived environments and stress levels. Participants are informed on the study options via the survey-landing page, receive a standardised overview of the study and give their informed consent. Except for participants requesting a smartphone, the entire survey process is automated, including registration, sending questionnaires and reminders via e-mails, downloading the app with instructions, sending login and password, starting the survey, sending encouragement e-mails throughout the mobile survey and closing the survey.

### Sampling and recruitment

Inclusion criteria require participants to be aged 18–65 years old, to reside in the Grand Duchy of Luxembourg and to be fluent enough in English, French or German to complete a 30 min questionnaire. Participants are recruited through convenience sampling using mixed approaches combining online methodologies such as social networks advertisements (ie, Facebook, Instagram), dissemination list of institutions and partners, a press release and unpaid news article, and offline approaches included flyers and posters disseminated in strategic locations, a radio interview and podcast and talks at national or local events (ie, Mental health weeks). The project calls on ambassadors to disseminate the recruitment call including national stakeholders, NGOs and citizen groups. Finally, as recommended,^59^ we use targeted sampling strategies to recruit underrepresented groups in our sample, including young adults, men and participants residing in municipalities of lower urbanity degree or municipalities of less advantaged socio-economic status. A total of 2000 participants is recruited over a maximum of 1 year for the online survey (eg, partial option), and a subsample of 200 participants is expected to engage in the mobile survey (eg, full option). To increase the response rate, we offer incentives as lotteries of twenty-four €100 vouchers for the full option and twelve €50 vouchers for the partial option, with a draw every 3 months.

### Procedures

#### Online survey: regular activities, environmental exposures and chronic stress

The online survey aims to identify regular activity patterns and related regular exposure patterns of participants to be investigated in relation to chronic stress. It includes two successive online questionnaires, each lasting 20 to 30 min.

##### Traditional web questionnaire

The participant is guided through a web-based questionnaire operated by the Eco–Emo Tracker application on the following dimensions: household composition, home characteristics, socio-demographics, stress and well-being, personality, quality of life and health behaviours, social support, perception of the neighbourhood, residential self-selection, and mobility and transportation. Detailed measures are provided in table 1.

**Table 1:** Constructs, variables and sources of questionnaires used in the online survey

Construct and variables	Items	Reference/source
Stress and well-being		
Chronic stress	10	Perceived stress scale – 10 items^89^
Stress Causes – General	1	Self-developed
Occupational stress	3	Canadian Forces Occupational Stress Questionnaire^90^
Financial stress – income needs	1	ABENA study^91^
Consumption of drugs/medication impacting stress	1	Adapted from the Canadian Community Health Survey – Mental Health (2012) - Santé Mentale (2012)
Perceived well-being	5	WHO-5 Well-being Index^92^
Quality of life & Health Behaviours		
General Mental and Physical quality of life	16	WHO- Quality of Life - Physical and psychological quality of life sub-index^93^
Sleep	4	Canadian Community Health Survey^94^
Physical Activity & Sedentary Behaviours	8	International Physical activity Questionnaire – short form^95^
Personality		
Personality traits	10	Big Five Inventory-10^96^
Mobility and transportation		
Driving licence and car availability	2	
Regular transportation mode	1	Adapted from the CURHA study^48^
Neighbourhood characteristics		
Neighbourhood stress	2	RECORD Cohort Study^97^
Neighbourhood insecurity	3	RECORD Cohort Study^97^
Neighbourhood disorder & violence	10	CURHA study^48^
Neighbourhood restorative environments	27	Social cohesion scale ; INTERACT Study ; Self-developed items adapted from Refs. 98,100
Neighbourhood self-selection	11	CURHA study ; self-developed^48^
Environmental preferences	2	Self-developed
Social support		
Social support from family and friends	6	Lübben Social Network Scale-6^101^
Social support in the neighbourhood	1	RECORD Cohort Study^97^

##### Map-based web questionnaire

**Figure 1:**
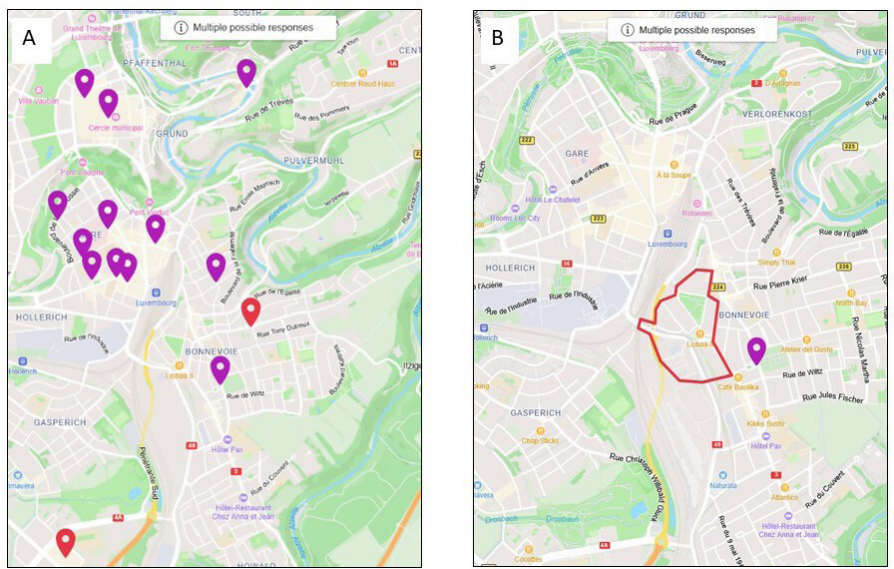
Geolocation of a participant’s activity space (**A**) and stressful area (**B**) via the VERITAS map-based questionnaire.

Participants’ regular mobility and activity patterns are then surveyed with the ‘Visualisation and Evaluation of Regular Individual Travel destinations and Activity Spaces’ (VERITAS) application^29^ implemented in the Eco–Emo Tracker application. Using the web-based mapping tool, participants are invited to report the location of up to 17 types of destinations they may engage in a typical week (figure 1A). It comprises the main residence, the workplace, shopping locations (eg, supermarkets, bakery and shopping centre), leisure locations (eg, sports or leisure-time physical activity location, park or other natural public space, food and drinks establishments), place of social visit (family/friends/colleagues), place where to drop or pick up people and other regular places. For each destination, participants are invited to report the frequency and average visit duration, with whom the activity is usually performed,^60^ and which main transportation mode is used to reach the destination. In addition, participants can indicate the areas near their place of residence that they perceive as stressful and report the reasons for their stress and indicate how frequently they pass them (figure 1B).

### Mobile survey: GEMA of daily activities, environmental exposures and momentary stress

The survey is supported by the FragMent app, tailored from Eco-Emo Tracker application^34^ for the need of the FragMent study. As the application containing all the options required for the FragMent survey is currently only available on Android OS, participants could choose to use their own Android smartphone if they had one or request a smartphone provided by the study team for the time of the survey (Samsung A21, Android 12). They receive clear instructions not to start the survey if they plan to be on holiday or to leave Luxembourg for more than 2 days during the data collection period. Participants are asked to charge the smartphone every night and not to disable the smartphone GPS as well as the survey notifications from the FragMent app. The day before the survey starts (eg, day 0), participants receive a short overview of the survey structure, tasks, type of questions and answer modalities (eg, slider, single/multiple choice questions and vocal task) to familiarise themselves with the app and mitigate stress due to the novelty of the survey.^61^

During the 15-day Ecological Momentary Assessment (EMA) phase, participants are prompted four to six times per day following recommendations on EMA protocol adherence.^62^ Four schedule-based prompts within a random component of 30 min are sent every 4 hours at 8 am, 12 pm, 4 pm and 8 pm to cover different activities, locations and environmental contexts.^14 63^ Each prompt is available for 1 hour and a reminder to complete the questionnaire is sent after 45 min. At each of the four schedule-based prompts, participants must complete a short questionnaire and a vocal task. Two additional prompts are event-based and conditionally triggered if participants are outdoors sometime between 09:30 am and 11:30, and between 17:30 and 19:30 to specifically collect data on the outdoor experience using a short questionnaire. The system detects the participant’s outdoor location using GPS information, including the number of satellites connected to the smartphone and precision indicators. No vocal tasks are requested on the event-based prompt to minimise outdoor noise interference. No reminders are sent for the event-based prompts, and their availability to the participants is limited to 30 min. The app continuously collects smartphone GPS-based locational data over 15 days, at a sampling rate of 5 s. Thanks to an automatic recognition of trips and stops, participants are further invited to fill in a transport and activity logs via the app. Both location data and answers to questionnaires are transmitted in real-time to the survey platform on servers exclusively located in Europe. During the 15-day monitoring period, the participants are kept informed of the ongoing collection of GPS and survey data by a permanent notification display about the data collection progress on their smartphone.

#### Measures

**Figure 2:**
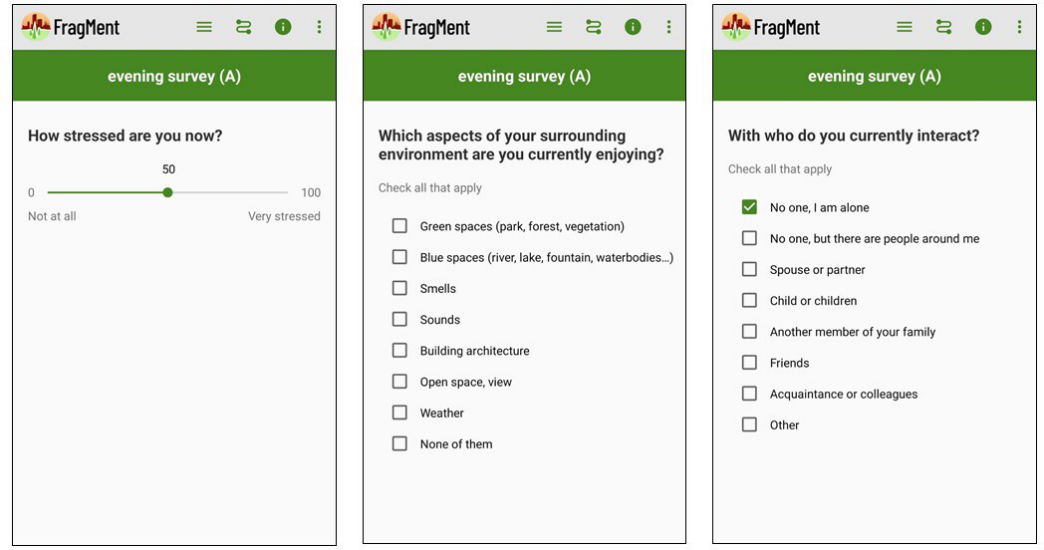
Example of FragMent core EMA questions.

**Figure 3:**
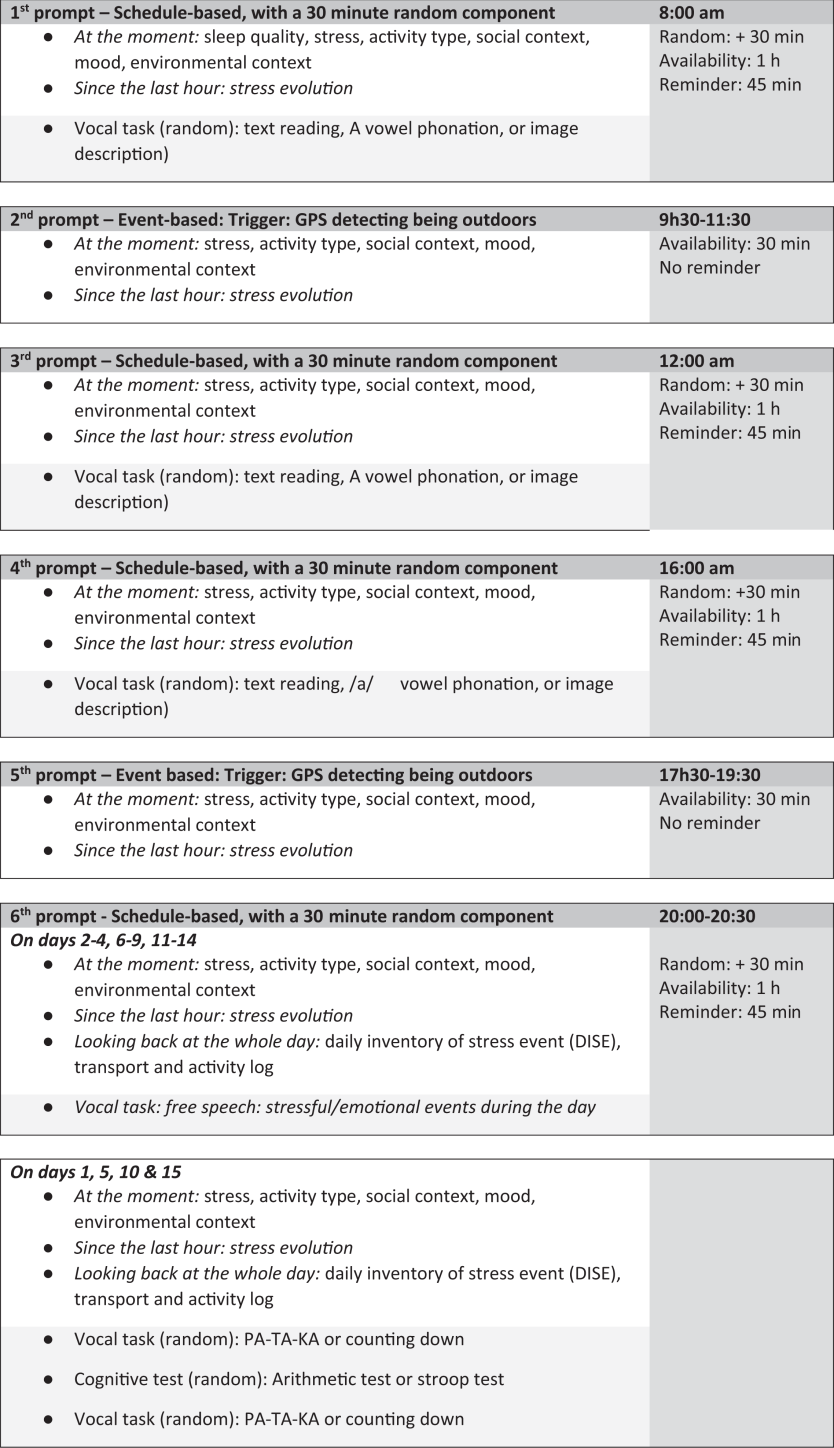
EMA design over 15 days.

The 6 prompts encompass 7 to 10 EMA questions and take on average 1 to 2 min to be completed. EMA questions appear in a randomised order between prompts to reduce participants’ burden and annoyance due to repetitiveness.^64^ A list of core items is identical across the prompts, while morning and evening prompts include specific items linked to sleep quality, stressors encountered during the day and an incentive to complete the transport and activity logs. Figure 2 presents an example of EMA core questions on the participant’s smartphone and figure 3 presents the EMA design over 15 days.

#### Sleep

Sleep quality is assessed every morning with an adapted version of the single-item sleep quality scale,^65^ ranging from 0 (terrible) to 100 (excellent).

#### Stress, stressful events and mood

Momentary stress is evaluated with one single item ‘how stressed are you now’, ranging from 0 (Not at all) to 100 (Very stressed).^66^ A second stress-related item assesses how the participant’s stress level has changed over the past hour, based on a 5-point Likert scale response option. Mood is evaluated with the Visual Analogue Mood Scale, ranging from 0 (Sad) to 100 (Happy).^62^ In the evening prompt, we use an adapted version of the Daily Inventory of Stressful Events^67^ as a checklist of potential stressors encountered during the day regarding seven contexts (eg, your kid(s), partner or close friend and work/study related context).

#### Vocal tasks for stress biomarkers

**Figure 4:**
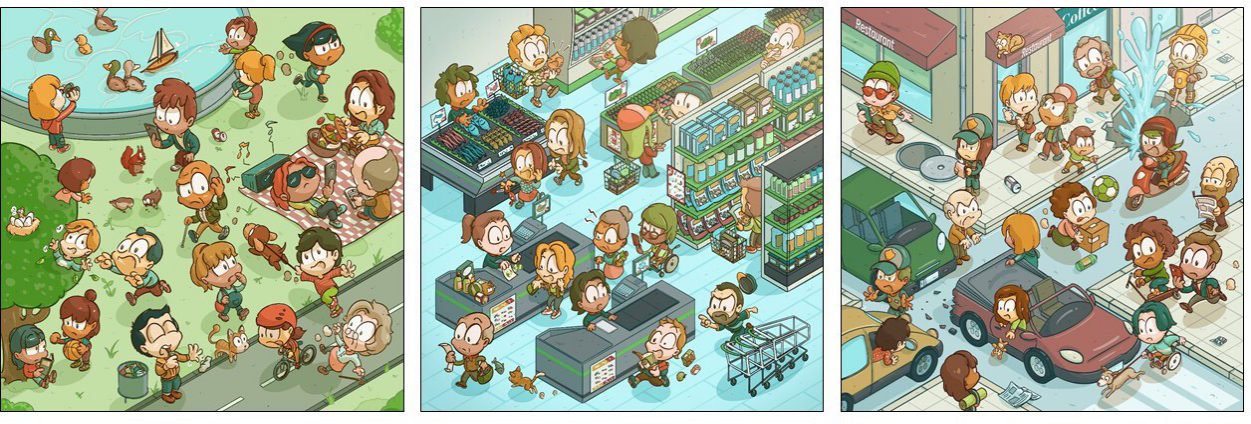
Comic-style images of daily life situations, created for the picture description tasks. Participants are instructed to describe the image or parts of the image for at least 20 s.

Six types of vocal tasks are developed to analyse voice recordings. On days 1, 5, 10 and 15 (figure 3, sixth prompt), two vocal tasks are implemented before and after the completion of a stress induction task to provide gold standards for detecting stress levels based on within-participant comparisons. We rely on two validated stressful tasks, including a timed (ie, 60 s) arithmetic task^68 69^ and a timed adapted version of the Stroop test.^50 70^ Due to the repetition of the same vocal task at a 60-s interval, it is important that these two tasks present low levels of habituation for the participant, which led to our choice to count down from 20 to 0 (task 1), and repeat PA-TA-KA as fast as possible for 10 s (task 2). The PA-TA-KA test was previously used to identify vocal biomarkers of neurological disorders,^71 72^ while counting is a common task for vocal biomarker identification.^50 51 73^ The other four types of vocal tasks are used as stand-alone tasks, comprising structured and unstructured tasks, and consisting of reading aloud a predefined text,^73^,^75^ describing an image,^51 76^ the /a/ vowel phonation^73^ (ie, saying ‘ahhhh’ as long as possible in one single breath) and free speech.^75^ Regarding the reading aloud task, three texts with a neutral (ie, the human right declaration as reported elsewhere), happy and sad tone (excerpts from The Little Prince, a widely translated and familiar/accessible text) have been selected to last approximately 20 s each. Regarding the image description, in collaboration with a comic designer, three images representing daily scenes in Luxembourg–a park, a supermarket and a busy street – have been designed according to a set of guidelines for vocal biomarkers identifications (figure 4).^77^

#### Activity and social interactions

At each prompt and via a single-option question item, participants have to specify the type of activity they were engaged in (eg, work/study, transport, shopping, sport, leisure, household chores, family care, etc.), as well as to indicate via a checklist the people they are interacting with at that moment (eg, no one - I am alone, no one - but there are people around me, spouse or partner, child or children, other family member, friends, acquaintance or colleagues, other).

#### Perceived environment

At each prompt and via two multiple-option question items, participants report elements of their surrounding environment they are currently enjoying (eg, greenspaces, blue spaces, smells, sounds, building architecture, open space/view, weather), and the ones that are currently a problem to them (eg, noise, crowdedness, pollution, traffic, incivilities, building facades in poor condition, weather).

#### Mobility

**Figure 5:**
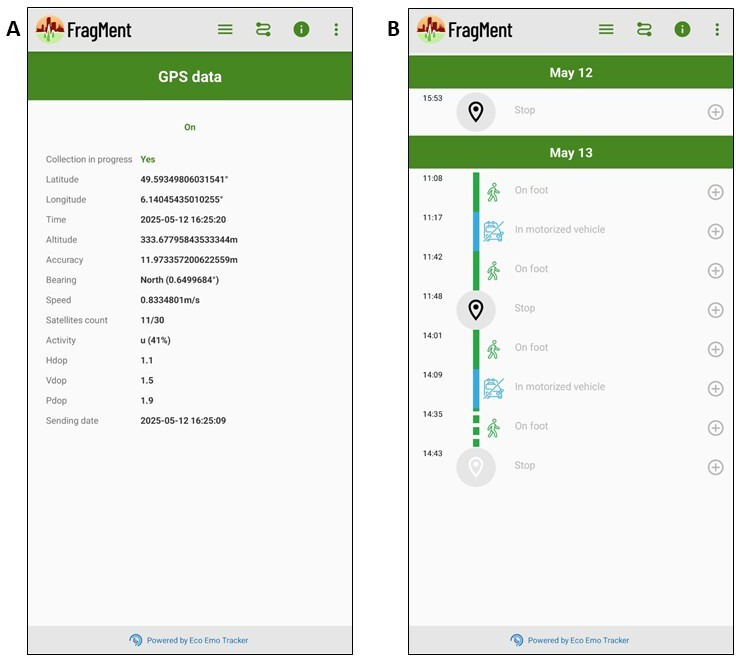
Example of FragMent mobility data (**A**) and transport and activity log (**B**). Participants are instructed to visualise, verify and complete their mobility and activity data every evening.

GPS-derived variables include timestamp, longitude, latitude, number of satellites connected, altitude, precision, direction, activity, Horizontal Dilution of Precision (HDOP), Vertical Dilution of Precision (VDOP), Position Dilution of Precision (PDOP) and speed (figure 5A).

### Transport modes and location type

A transport and activity log is automatically created as the participant travels from one location to another. GPS-based algorithms automatically detect clusters of GPS points that are defined as stops and more dynamic segments that are defined as trips. For each trip segment, algorithms further classify a transport mode (eg, on foot, bicycle, motorised transportation mode, subway). This log is available to the participant at any time of the survey via a dedicated screen of the app (figure 5B) with the possibility to visualise, correct and complement the predicted transport mode by specifying their actual mode of transport, indicating the type of location visited (eg, home, work/study place, gastronomy, leisure, shopping, nature, family/friend’s place, etc.) and whether this activity location was performed inside or outside. As a reminder, participants are invited every evening to verify and complement their own mobility and activity data.

### Environmental exposure measurements and modelling

The regular, daily and momentary environmental contexts will be assessed objectively using environmental indicators derived from a Geographic Information System (GIS). Environmental indicators will relate to the socio-demographic, built and natural environments. Regular destinations reported with the map-based questionnaire and GPS data collected through the FragMent app will be enriched with the GIS data. Exposure will be measured using standard circular street network buffers of various sizes along the GPS tracks (eg, 50 m, 100 m and 200 m)^78^ as well as around regular destinations collected through the VERITAS map-based questionnaire (500 m, 800 m 1000 m), aiming at representing the relevant exposure areas to be tested in sensitivity analyses. Finally, time-weighted exposure measurements^79^ (ie, kernel density estimation models, density ranking and point overlay) accounting for time spent at different locations and during travel will be further investigated and compared with unweighted exposure models.

### Analytical approach and power analysis

#### Identification of vocal biomarkers of stress

Identification of vocal biomarkers of stress includes the following steps^51^: (i) audio preprocessing, including resampling, normalisation, noise reduction, framing and windowing the data; (ii) audio feature extraction using the library DisVoice (https://disvoice.readthedocs.io/en/latest/reference.html), including glottal, phonation, prosody, articulation and phonological features; (iii) audio feature selection (eg, selection of “explainable feature” such as mean and SD) and dimensionality reduction (eg, via principal component analysis) aimed at selecting the most relevant features for the prediction of stress; (iv) automatic classification of stress based on machine learning algorithms (eg, XGBoost, Support Vector Machine); and (v) computation of performance metrics including accuracy, specificity and sensitivity metrics. In order to avoid systematic bias toward minorities (groups under-represented in the dataset) in the ability to accurately classify stress, algorithms have been pretrained on the Colive Voice dataset, an international and anonymous cohort aiming at detecting vocal biomarker candidates of numerous health outcomes based on vocal tasks from participants located worldwide.^73 74 80^

#### Relationships of environmental exposure and activity patterns, with momentary, daily and chronic stress

Multivariate adjusted models will be used to test the association between regular exposure patterns, regular activity patterns and chronic stress. Multilevel mixed adjusted models will be used to account for the repeated sampling design (eg, multiple measurements per participant) in the association between momentary/daily environmental exposures, activity patterns and momentary/daily stress. Social inequalities in stress will be examined by adding interaction terms to test the moderating effect of gender and socio-economic status on the relationships between exposure patterns, activity patterns and stress. Specific attention will be paid to minimise potential residential self-selection bias^81^ (ie, by controlling for individual preferences people had when moving in their residential neighbourhood) and selective daily mobility bias^82 83^ (ie, by considering truncated activity spaces measures, comparing actual path between locations to the shortest path, etc.). As our sample may deviate from the general population, we will use survey weights.

#### Power analysis

The following elements were considered to calculate the sample size. First, an estimated 10% of the participants completing the survey is expected to engage in the mobile survey (full option).^78^ A minimum of 200 participants is needed to ensure sufficient variability in terms of demographics and feasibility of the moderation analyses by social groups. Second, participants are followed over 15 days to ensure observing sufficient potential within-person variability. Third, a high power is needed to account for variability in effect size by environmental factor. Fourth, four to six micro-questionnaires per day will be sent to participants. A completion rate of 66% is expected.^62^ Given these considerations, Monte Carlo simulations were conducted using the R package SIMR^84^ to determine the power of an analysis with the following characteristics: a generalised linear mixed model for a binomial outcome (‘high stress’ vs ‘low stress’) with 17% of observations estimated to be ‘high stress’^28^; a continuous, within-person varying predictor, a+2% expected change in high-stress prevalence related to an increase of one SD in the predictor^14^ and two random effects controlling for the nested structure of the data (ie, repeated measurements per individuals, themselves nested by municipality of residence. After 1000 simulations, the results indicated a power of 99.80% (95% CI: 99.28, 99.98). Despite the very high estimated power, the number of participants will be maintained at 200 for the reasons stated above.

While the sample size of 200 participants was determined through power analysis, previous studies with similar designs have successfully drawn comparisons between demographic groups using sample sizes ranging from 100 to 250 participants.^17 85^ These studies included neighbourhood-level comparisons and incorporated sociodemographic variables in their models. In our study, we further enhanced this approach by employing targeted recruitment strategies to ensure inclusion of underrepresented groups, aiming for variability in age, gender, socio-economic status and residential environment.

### Pilot study

The feasibility of the study has been evaluated in a pilot study (n=12), performed in three languages (ie, English, French and German). Participants were recruited via snowball sampling and had to comply with the study eligibility criteria. For practical reasons, only the first traditional web-based questionnaire and the 15-day GEMA survey were evaluated in this pilot phase. A description of the participants’ socio-demographics and stress levels is provided in table 2.

**Table 2:** Descriptive statistics of the pilot sample (n=12)

	Percent (n)	Mean (SD)	Median	IQR
Age		42 (12.4)	41	21.5
Languages				
English	33 (4)			
French	42 (5)			
German	25 (3)			
Women	58 (7)			
Living alone	50 (6)			
Education level				
No Education to General secondary education	8 (1)			
Vocational or technical secondary education to Bachelor’s degree	8 (1)			
Bac+4 or equivalent to Doctorate	84 (10)			
Smartphone request to conduct the study	66 (8)			
Physiological quality of life (4-20)		14 (2.1)	15	2.1
Psychological quality of life (4-20)		15 (2.1)	15	3.7
Perceived chronic stress (0–40)		22 (7.6)	23	8.5

Overall, 12 participants answered 401 questionnaires over the 15-day period, among which 90% (361 questionnaires) were schedule-based and 10% (40 questionnaires) were event-based, triggered by the outdoor location of the participant. Out of a total expected number of 60 scheduled-based questionnaires, a participant answered on average 33.4 questionnaires, with a median of 13 days and a minimum of one answer per day. The distribution of the response rate over the day is similar in the morning, at noon, in the afternoon and in the evening (range: 49.4 %–50.5 %). Most of the answered prompts were filled within 45 min (84.3%) (ie, without reminders).

Regarding the vocal tasks, 81.8% of the vocal recordings were exploitable to analyse vocal biomarkers of stress, including clean (64.3%) and slightly noisy (17.5%) recordings. Empty (2.1%), noisy (11.7%), wrong task (0.5%), and other' (ie, people whispering, microphone covered, etc.) (3.9%) recordings will not be analysed.

Participants reported low to medium momentary stress levels (mean (sd)=25.2 (21.5)), which stayed stable over the last hour in 39.4% of cases, increased in 25.2% of the cases and decreased in 35.4% of the cases. Stressors identified in the evening via the adapted version of the Daily Inventory of Stressful Event (DISE) most frequently fell under *‘nothing stressful’* (47.2%), followed by stressors experimented in the *‘Work/study or work/study-related context’* in 29.2% of cases.

Regarding the perceived environment, participants identified no problems in their surrounding environment in most cases (79.8%), while they often enjoyed the open space/view (26.4%), the weather (24.2%) and the presence of greenspaces (19.5%). Most prompts were answered at work (27.9%), while resting (14.7%), eating (13.0%) or during leisure activities (12.7%). Finally, 61.3% of the prompted were answered while being alone, 15% in the company of a spouse/partner and 10% in the company of children. A complete description of participants’ responses is provided in table 3.

**Table 3:** Descriptive statistics of the 15-day mobile survey

	Percent (n)	Mean (SD)	Median	IQR
Characteristics and adherence to the EMA survey design				
Pct. of answered EMA prompts by trigger (n=401)				
Schedule-based trigger	90.0 (361)			
Event-based (being outdoor) trigger	10.0 (40)			
Compliance rate of schedule-based prompts	50.1 (361)			
Nb. of answered EMA prompts per participant		33.4 (12.9)	25	23
Nb. of days with at least one answered EMA prompt per participant		12.4 (2.8)	13	2
Compliance rate of schedule-based EMA-prompt across the day				
Morning (08:00–09:00)	50.0 (90)			
Noon (12:000–13:00)	50.5 (91)			
Afternoon (16:00–17:00)	50.5 (91)			
Evening (20:00–21:00)	49.4 (89)			
Nb. of answered EMA prompts after reminder (45 min)	15.7 (60)			
Vocal tasks				
Compliance rate per vocal tasks				
All	50.0 (384)			
A Vowel phonation	52.4 (44)			
Free speech	50.0 (66)			
Image description	49.7 (113)			
Read-out-loud	50.5 (115)			
Count down	41.7 (20)			
PA-TA-KA task	54.2 (26)			
Quality of vocal recordings				
Clean	64.3 (247)			
Empty/silence	2.1 (8)			
Noisy	11.7 (45)			
Slight Noise	17.5 (67)			
Wrong task	0.5 (2)			
Other	3.9 (15)			
Stress and mood				
Momentary stress (0–100)		25.2 (21.5)	20	27
Stress evolution during the last hour				
Increased	25.2 (101)			
Not changed	39.4 (158)			
Decreased	35.4 (142)			
Mood (0–100)		71.2 (18.5)	71	38
Daily inventory of stressful events				
Your kid(s), partner or close friend	10.1 (9)			
A task you were performing	6.7 (6)			
While travelling	4.5 (4)			
Work/study or work/study-related context	29.2 (26)			
Home-related event	3.4 (3)			
Your health	4.5 (4)			
Other	4.5 (4)			
Nothing stressful	42.7 (38)			
Perceived environment, activities and social company				
Problems in the surrounding environment				
Noise (car, construction work, etc.)	7.0 (28)			
Crowdedness	2.2 (9)			
Pollution, smells	1.5 (6)			
Car traffic	4.2 (17)			
Feeling of insecurity (people, blind wall, lack of lighting, etc.)	0.3 (1)			
Physical decay (poorly maintained infrastructure, garbage, graffiti, etc.)	0 (0)			
Weather	8.7 (35)			
None of them	79.8 (320)			
Enjoyment of the surrounding environment				
Green spaces (park, forest, vegetation)	19.5 (78)			
Blue spaces (river, lake, fountain, waterbodies…)	0.8 (3)			
Smells	7.5 (30)			
Sounds	12.7 (51)			
Building architecture	6.0 (24)			
Open space, view	26.4 (106)			
Weather	24.2 (97)			
None of them	42.4 (170)			
Activities				
Work/study	27.9 (112)			
Transportation (car, train, bus, bicycle, walking…)	9.2 (37)			
Shopping	1.7 (7)			
Sports	3.2 (13)			
Leisure activities	12.7 (51)			
Household chores	8.5 (34)			
Family care activities	3.5 (14)			
Self-care activities	5.5 (22)			
Eating	13.0 (52)			
Rest	14.7 (59)			
Social company				
No one, I am alone	40.6 (163)			
No one, but there are people around me	20.7 (83)			
Spouse or partner	15.0 (60)			
Child or children	10.5 (42)			
Another member of your family	1.2 (5)			
Friends	8.2 (33)			
Acquaintance or colleagues	9.5 (38)			
Other	1.2 (5)			

After the completion of each survey, participants responded to feedback questions adapted from Eisele (2022)^86^ regarding perceived burden, ease of use, instructions, and careless responding, and provided additional feedback on their overall experience in open text sections (online supplemental table 1). Participants reported a moderate to high enjoyment of completing the survey, which was generally evaluated as rather easy to complete. Using the app was assessed as low to moderately stressful. The survey was evaluated as moderately tiring, irritating and disturbing of one’s everyday life. The vocal tasks were also moderately stressful and irritating, and participants reported a good to very good understanding of the instructions to perform the vocal tasks. The participants' perception of moderate support from the research team during the survey was addressed in the study by sending four encouragement e-mails over the 15-day monitoring period to help maintain their motivation and provide support when needed.

### Participant and public involvement

None.

## Discussion

FragMent is the first study combining the investigation of momentary, daily and chronic stress, measured objectively and subjectively, in relation to immediate, daily and regular exposures to environments, and activity patterns. While FragMent will advance existing knowledge on environmental determinants of stress, it departs from traditional neighbourhood and health studies by accounting for individual mobilities in exposure assessment in many conceptual and methodological ways.

The study examines the in-depth effects temporarily in neighbourhood effects on stress by disentangling exposure patterns into exposure durations, frequencies and temporal configuration over the course of the day (ie, sequences, temporal clusters, etc.).^42^ Notably, a better understanding of the effect of exposure temporalities in stress may translate into a better estimation of social inequalities in stress. Indeed, more vulnerable social groups may be exposed more frequently to stressful environments, spend more time therein and be less able to alternate sequences of exposures from stressful to more restorative environments. Not accounting for exposure temporality may thus result in a systematic misestimation of social inequalities in stress.

FragMent is the first study to combine a GEMA design with voice technology, considered today as one of the most promising sectors to identify symptoms in a non-invasive way,^51^ in order to detect biomarkers of stress. Observational or clinical studies embracing voice technology typically only include multiple recordings at one point in time or a single vocal recording prestressor and posttressor exposure. Compared with these studies, the repeated daily vocal sampling (four times per day) over 15 days of our study will produce a unique dataset (expected 7920–12000 vocal recordings), rich in multiple vocal tasks (eg, image description, text reading, etc.). FragMent vocal tasks further include the completion of four vocal tasks prestress and poststress induction tasks (ie, two arithmetic tasks and two Stroop tests). Although the level of difficulty of the tasks was not adjusted on the participant’s capabilities, this aimed to provide four gold standards over 15 days for detecting stress levels based on within-participant comparisons. Overall, the FragMent dataset will open up new opportunities for in-depth investigations of vocal stress biomarkers, including within and between participant variations thereof and tied to varying environmental contexts. Furthermore, the ecological nature of the recordings helps to better identify vocal biomarkers in real stressful situations, as compared with clinical studies relying on ‘artificial‘ stressors. Finally, to our knowledge, FragMent is the first study to investigate the effect of environmental stressors on vocal biomarkers.

The 15-day follow-up provides a unique opportunity to observe full variability in stress experiences and associated environmental exposures and activities across various spatial and time-related contexts (ie, weekends vs weekdays; mornings vs noon vs afternoons vs evenings). Indeed, a recent review of GEMA studies for establishing new reporting guidelines reported only 4 studies out of 20 with a longer monitoring period ranging from 30 to 60 days.^64^ The 15-day monitoring period further addresses the relative lack of accuracy in capturing participants’ full activity space in GPS studies below the recommended 2 weeks threshold.^87^

FragMent uses the latest technological developments in mobile sensing and EMA software to not only sample participants’ stress experiences in various time-related contexts, environments and activities but also specifically target their outdoor experiences by triggering questionnaires based on their location. While the outdoor triggering of questionnaires, based on GPS information (ie, number of satellites connected and precision indicators), may be sensitive to the proximity to large windows or to urban canyons, we believe that collecting information on environmental exposures via a window, rather than a proper outdoor exposure, would still provide valuable insights on the participant’s visible surrounding environment.

The Eco–Emo tracker GEMA application enables us to produce and investigate acute and innovative indicators of participants’ sequences of activities in relation to their stress levels by combining momentary questionnaires on activities and social context, continuous GPS tracking and associated time-stamp and automatically processed daily visualisations of the participants’ timelines of stops, trips and transport modes to be verified, corrected and completed by the participant. However, building accurate sequences of exposures and activities remains challenging due to signal loss, incomplete or unverified timeline data and response rate to daily questionnaires.

A further strength of FragMent is its fully automated survey process based on a prewritten study script embedded in the application. This enables a large-scale GEMA study with national coverage across a wide adult age range (18–65 years old), while requiring minimal technical and human resources. Despite its feasibility and scalability, the multisurvey components of FragMent with a 15-day follow-up come with challenges to foster high completion rates and to limit the attrition at each step of the survey period.

The pilot study registers a 50% compliance rate, which is acceptable compared with GEMA studies reporting between 50% to 100% compliance rates.^64^ However, the addition of vocal tasks precludes a full comparison with more traditional (G)EMA studies, as the nature of the vocal task itself implies the need to speak aloud on the phone, which may not always be perceived as feasible by the participant in every context. The mismatch between the compliance rate observed in the pilot study and the expected compliance rate considered in the power analysis (66%) may result in a lower analytical power. However, pilot studies should not be used to inform power calculation due to their limited samples,^88^ but should be used to evaluate the feasibility, which was confirmed here. Furthermore, the estimated power from 200 participants engaged in the mobile survey was very high (99.8%), indicating that foreseen analyses may not suffer from a slightly lower response rate. Finally, the survey design was improved after the pilot to maintain participant adherence to the protocol with encouraging e-mails sent over the monitoring period.

Regarding causal inference, the study has developed some questionnaire items and methods to control for confounding linked to residential self-selection and selective daily mobility. In addition, the 15-day GEMA design allows for the examination of micro-longitudinal within-person stress-environment associations. Notably, the study will be able to investigate temporal sequences with environmental exposures assessed prior in time to stress evaluation. With these approaches, we will attempt to improve as much as possible the quality of causal inference from our data.

Finally, the possibility that the mobile survey and associated vocal tasks are experienced as stressful by the participant cannot be ruled out. To cope with this bias, participants are asked to experience the typical survey questions and vocal tasks 24 hours before the start of the survey to reduce the effect of surprise linked to the first vocal recording. The 15-day data collection will further enable us to perform some sensitivity analyses by comparing the stress levels with and without the first one or 2 days of the survey, during which the participant may be more stressed to miss a questionnaire or perform a vocal recording.

### Ethics and dissemination

The FragMent protocol was approved by the Research Ethics Committee of the Luxembourg Institute of Socio-Economic Research (LISER) (reference: LISER REC/2021/024.FRAGMENT/4-5-9-10). Participants received detailed information on the entire study procedure and all signed an informed consent form before entering the study. The personal data collected during the study will be processed and stored in accordance with the General Data Protection Regulation (GDPR). Only the research staff in charge of the data collection will have access to direct participant identifiers (ie, name and e-mail address). The data will be pseudonymised and principles of data separation will be applied to mitigate the risk of re-identification. Data will be stored on secured servers and only named researchers will have access to the data. Access to data on LISER’s secure servers is conducted within a controlled environment that excludes internet connectivity and restricts the use of any software not previously approved by the LISER Data Management Team. Prior to publication, the results will be checked for privacy (ie, absence of potential re-identification of participants) by a LISER data manager. Pseudonymised data will be stored for 10 years after the end of the project for replication purposes.

The core dissemination of the study findings will be the publication of peer-reviewed articles in fully open-access journals. Participatory workshops will inform project partners and local and national stakeholders about the results over the different project phases of the project (eg, survey design, preliminary results, final results interpretation and implications for local/national policies). Videos and comic strips will inform the lay audience about key findings, in addition to press releases and social media posts. All FragMent-related publications and outputs can be downloaded at www.fragmentproject.eu.

## Supplementary material

10.1136/bmjopen-2025-105499 online supplemental file 1
