# Supplementary material for: Geographic environments, daily activities and stress in Luxembourg (the FragMent study): a protocol combining map-based questionnaires, geographically explicit ecological momentary assessment and vocal biomarkers of stress
Source: BMJ Open. 2025 Sep 2;15(9):e105499. doi: 10.1136/bmjopen-2025-105499 (PMC12406917; doi:10.1136/bmjopen-2025-105499)
Supplement: online supplemental file 1 [file bmjopen-15-9-s001.docx]

**Supplementary Table 1. Evaluation of the 15-day mobile survey and repeated vocal sampling**

| **Items** | **Mean (SD)** | **Median (IQR)** |
| --- | --- | --- |
| **Perceived burden** | | |
| Answering to the questions disrupted my everyday life. | 3.6 (1.4) | 3.5 (3.0) |
| The questionnaires on the phone stopped me from doing my usual activities. | 2.6 (1.6) | 2.0 (2.5) |
| I found it embarrassing when the alarm sounded around other people | 2.2 (1.4) | 2.0 (2.0) |
| I enjoyed using the app | 4.3 (1.8) | 4.0 (2.2) |
| I found it stressful to use the app | 2.8 (1.7) | 2.5 (3.0) |
| My motivation to respond to the beeps decrease during the weeks | 4.3 (1.8) | 5.0 (1.5) |
| It was tiring to take part in this survey | 3.5 (2.0) | 4.0 (3.2) |
| I get irritated while filling in the survey | 3.1 (1.8) | 3.0 (2.5) |
| Performing the daily vocal tasks was stressful to me | 4.2 (2.2) | 4.5 (3.5) |
| I get irritated while performing the vocal tasks | 3.9 (2.2) | 4.0 (4.0) |
| **Instructions** | | |
| I understood all the vocal tasks | 5.5 (1.4) | 6.0 (3.0) |
| **Ease of use** | | |
| The questionnaires on the phone were easy to complete | 5.7 (1.2) | 6.0 (1.2) |
| At times, I had to rush to complete the questionnaires on the phone on time. | 4.2 (2.1) | 5.0 (2.5) |
| I found it easy to remember to carry the phone with me during the time of the survey | 3.6 (2.2) | 3.0 (4.0) |
| **Engagement** | | |
| The “Day 0 – user guide” I received at the beginning of the survey was adequate to use the app for two weeks | 5.8 (1.1) | 6.0 (2.0) |
| I felt supported by the researchers during the survey | 4.1 (1.8) | 4.0 (1.2) |
| I filled out the questions without thinking about myself | 2.7 (1.6) | 2.5 (3.0) |
| I responded carelessly to the questions | 2.7 (1.8) | 2.0 (1.2) |

*Note: response scales ranged from 0 (Not at all) to 7 (Very much).*
